# From feeling depressed to getting diagnosed: Determinants of a diagnosis of depression after experiencing symptoms

**DOI:** 10.1177/00207640241303038

**Published:** 2024-12-26

**Authors:** Barbara Stacherl, Theresa M Entringer

**Affiliations:** 1 German Institute for Economic Research (DIW Berlin), Germany; 2 Department of Psychology, University of Greifswald, Germany

**Keywords:** Depressive disorder, depression symptoms, diagnosis, personality, spatial access

## Abstract

**Background:**

Receiving a formal diagnosis for a depressive disorder is a prerequisite for getting treatment, yet the illness inherently complicates care-seeking. Thus, understanding the process from depression symptoms to diagnosis is crucial.

**Aims:**

This study aims to disentangle (1) risk factors for depression symptoms from (2) facilitators and barriers to receiving a diagnosis *after* experiencing depression symptoms.

**Method:**

We used data from the German Socio-Economic Panel. Within a sample of 40,238 individuals, we investigated factors predicting depression symptoms, assessed with the SF-12 Mental Component Summary score. Additionally, within a subsample of 3,444 individuals with depression symptoms, we analyzed factors associated with receiving a first-ever diagnosis in the subsequent year. These factors included health status, demographics, socioeconomic characteristics, personality traits, and health infrastructure.

**Results:**

Depression symptoms were associated with chronic physical conditions, female gender, middle age, living alone, fewer close friends, being unemployed or not working, lower income, lower agreeableness, conscientiousness, or extraversion, and higher neuroticism. Additionally, poorer overall mental and physical health, female gender, older age, unemployment, and neuroticism were positively associated with receiving a formal diagnosis. Access to general practitioners and psychotherapists was not associated with receiving a formal diagnosis.

**Conclusions:**

Our results replicated previous research on risk factors for depression symptoms. Moreover, some risk factors for experiencing symptoms (female gender, middle age, unemployment, and higher neuroticism) subsequently also facilitated receiving a formal depression diagnosis. Thus, this study underscores the importance of considering the chronological sequence in the process from depression symptoms to diagnosis.

## Introduction

Depressive disorders rank prominently as a cause of years lived with disability globally, highlighting their widespread prevalence (Vos et al., 2015). For instance, about one fifth of adults in Europe are believed to experience current symptoms of depression at any given moment (Arias-de la Torre et al., 2021). Moreover, in high-income countries, the average lifetime occurrence of depressive disorder is estimated to be around one in seven individuals (Hammen & Watkins, 2018).

However, depression symptoms often remain untreated (Handy et al., 2022), leading to adverse outcomes such as heightened depression severity (Hung et al., 2017), reduced remission rates (Bukh et al., 2013), and an elevated risk of suicide (Kraus et al., 2019). Thus, ensuring appropriate treatment for those with depressive disorders is paramount. Obtaining a formal diagnosis is a prerequisite for such treatment. However, the illness itself makes it inherently difficult to seek care.

Hence, it is crucial to gain deeper insights into the determinants of receiving a formal diagnosis of depressive disorder after encountering symptoms of depression. Therefore, our research investigates determinants (1) of experiencing depression symptoms, and (2) of receiving a formal diagnosis *after* experiencing symptoms, focusing on health status (e.g. chronic physical conditions, overall physical health), demographics (e.g. age, gender), socioeconomic characteristics (e.g. employment status, income), personality traits (e.g. the Big Five), and health infrastructure (e.g. spatial access to general practitioners).

### Pathway from depression symptoms to receiving a formal diagnosis

According to the ‘access to health care’ framework proposed by Levesque et al. (2013), we assume that there is a connection between depression symptoms and formal diagnosis via an access pathway (Supplemental Figure S1). This pathway starts with individuals recognizing a need for healthcare, indicated by experiencing depression symptoms, and concludes with individuals receiving a formal diagnosis of a depressive disorder (and subsequent treatment), representing a consequence of successful access to healthcare. At the start of this pathway, depression symptoms may be influenced by various risk factors pertaining to health status, demographics, socioeconomic characteristics, and personality traits. Following the onset of depression symptoms, factors pertaining to health status, demographics, socioeconomic characteristics, personality traits, and health infrastructure may act as facilitators or barriers along the pathway to receiving a formal diagnosis.

Most of the risk factors listed above have been found to predict depression symptoms (for a comprehensive overview, see Hammen & Watkins, 2018; Remes et al., 2021). For example, individuals with a chronic physical condition are more susceptible to depression (Steptoe, 2006). Moreover, women are almost twice as likely as men to report depression symptoms (Salk et al., 2017). Additionally, individuals with lower socioeconomic status have a higher likelihood of developing depression symptoms (Lorant, 2003), and individuals with higher neuroticism are more likely to experience depression symptoms than individuals with lower neuroticism (Orth & Robins, 2013).

Some extant research has also pinpointed factors influencing the likelihood of receiving a formal diagnosis and following treatment when depression symptoms are present. For instance, individuals with chronic physical conditions are more prone to seek treatment for depression, irrespective of depression symptom severity (Araya et al., 2018). Moreover, women with depressive disorders are more inclined to seek professional help compared to men (Roberts et al., 2018). Thus, while gender differences exist in how depression symptoms are perceived and expressed (Martin et al., 2013), disparities also emerge in care-seeking behavior between genders (Möller-Leimkühler, 2002). Additionally, higher socioeconomic status (Vigo et al., 2022) and higher neuroticism (Goktan et al., 2022) correlate with an increased likelihood of diagnosis and treatment. Finally, longer distances to healthcare facilities serve as barriers to treatment, leading to delayed care-seeking (Desai et al., 2023).

Thus, previous research has provided valuable insights into risk factors for depression symptoms as well as facilitators and barriers to receiving a formal diagnosis. However, previous research has been constrained by at least two limitations. First, these studies often assessed mental health service use simultaneously with depression symptoms (e.g. Araya et al., 2018; Vigo et al., 2022). By neglecting the time sequence of events, these studies were not able to fully disentangle factors predicting depression symptoms from factors predicting receiving a formal diagnosis. Second, despite theoretical indications suggesting that limited spatial access to (i.e. distance to and availability of) health infrastructure could impede care acquisition (Levesque et al., 2013), previous studies largely ignored this aspect. For instance, individuals may face barriers to care when they have limited nearby physicians or encounter lengthy wait times for appointments. While spatial access to the health infrastructure has been linked to health outcomes in various clinical domains (e.g. in gynecology, Araujo et al., 2017; and oncology, Wan et al., 2012), its role within the realm of depressive disorders remains unexplored.

### The present study

The present research has two aims. First, we aim to replicate previous research on risk factors for experiencing depression symptoms in a large representative sample from Germany, examining health status (e.g. chronic physical conditions, overall physical health), demographics (e.g. age, gender), socioeconomic characteristics (e.g. employment status, income), personality traits (e.g. the Big Five), and health infrastructure (e.g. spatial access to general practitioners). Second, we aim to identify facilitators and barriers to receiving a formal diagnosis of a depressive disorder *after* experiencing depression symptoms, again examining health status, demographics, socioeconomic characteristics, personality traits, and health infrastructure. Thereby, we aim to disentangle factors that aid or impede diagnosis from those that contribute to an individual’s initial symptoms. We rely on longitudinal data from the German Socio-Economic Panel (GSOEP). Using the Mental Component Summary (MCS) score from the 12-item Short-Form Health Survey (SF-12; Ware et al., 1998) as a screening tool, we evaluate depression symptoms in the general German population (Vilagut et al., 2013).We then examine determinants of these depression symptoms, that is, risk factors of depression symptoms (Aim 1). Subsequently, for a subset of individuals with symptoms of depression, we track whether they report receiving a first-ever formal diagnosis of depressive disorder in the *following* year. We use this to examine determinants of receiving a diagnosis of depression after experiencing symptoms, that is, facilitators and barriers to obtaining a formal diagnosis (Aim 2).

## Methods

### Data

We relied on data from the German Socio-Economic Panel (GSOEP), a nationally representative household panel study conducted annually since 1984. This study includes individuals aged 17 years and older and screens for depression symptoms within the general population, while also querying respondents about specific diagnoses, such as depressive disorder (Goebel et al., 2019). Notably, the GSOEP contains all essential individual-level variables related to health status, sociodemographic characteristics, and personality traits for our research purposes. To assess health infrastructure, we integrated information on the location and capacity of physicians from the National Association of Statutory Health Insurance Physicians (Kassenärztliche Bundesvereinigung, KBV), which we combined with census population data to evaluate individuals’ spatial access to physicians (see below for details).

### Samples

We created two samples for our analyses. The first sample used GSOEP data waves 2010, 2014, and 2018 as base years in which depression symptoms were measured. We chose these waves because all predictors of interest were either measured in these years or the preceding year. For the sample used to (1) investigate risk factors for depression symptoms (*symptom sample*), we applied no additional selection criteria. For the sample used to (2) investigate facilitators and barriers to receiving a formal diagnosis (*diagnosis sample*), we applied two more selection criteria. First, we included only individuals who screened positive for depression symptoms (Vilagut et al., 2013). Second, we included only individuals who reported never having received a formal depressive disorder diagnosis prior to screening positive for depression symptoms. In cases where an individual screened positive for depression symptoms in several subsequent years, we retained only one observation (randomly selected; for details see Supplemental Table S2). In all instances where covariates were missing, we employed multiple imputation techniques (Buuren & Groothuis-Oudshoorn, 2011). This procedure resulted in a *symptom sample* containing *N* = 40,238 individuals (and a total of 68,222 observations) and a *diagnosis sample* containing *N* = 3,444 individuals.

### Measures

Table 1 provides an overview of all covariates included in the present research, together with their survey questions, response ranges, and time of assessment. All covariates were measured either in the same year as the depression symptoms or in the preceding year (see also Supplemental Figure S2). Importantly, the formal diagnosis was measured in the year after depression symptoms were measured.

**Table 1.** Covariates included in the present research.

Variable	Variable type	Range	Measurement	Surveyed	Assessed
Mental health status	Numeric	0–45.6	Mental health status was assessed with the absolute value of the MCS score. The MCS is constructed to range from 0 to 100 with higher scores reflecting better physical health. In the analysis, MCS score values could range only from 0 to 37.2, as we included only individuals with an MCS score below the screening cutoff in our sample.	Biennially (since 2002)	In base years 2010, 2014, 2018
Physical health status	Numeric	0–100	Physical health status was assessed with the absolute value of the PCS score. The PCS is constructed to range from 0 to 100 with higher scores reflecting better physical health.	Biennially (since 2002)	Same year as depressions symptoms
Chronic physical condition	Binary	No chronic physical condition (0), Chronic physical condition (1)	Chronic physical condition was assessed with respondents’ self-reports of doctors’ diagnoses of common diseases. We generated a binary variable indicating whether an individual had ever been diagnosed with at least one of cancer, cardiopathy, diabetes, or stroke.	Biennially (since 2009)	Year prior to depression symptoms
Age	Categorical	<30, 30–44, 45–59, 60–74, and 75+	Age was recorded for the year when symptoms were measured. We generated five age groups to capture a possible non-linear relationship.	Annually	Same year as depressions symptoms
Gender	Binary	Male (0), Female (1)	Question: ‘Are you: male? female?’	Annually	Same year as depressions symptoms
Migration background	Binary	No direct migration background (0), Direct migration background (1)	Migration was assessed as a direct migration background, that is, having been born outside of Germany.	Annually	Same year as depressions symptoms
Household composition	Binary	Living with others, Living alone	Household composition was assessed with a measure covering eight categories. We regrouped it into two categories (living with others, living alone) for this study.	Annually	Same year as depressions symptoms
Number of close friends	Numeric	0+	Question: ‘What would you say? How many close friends do you have?’	Irregularly (2003, 2008, 2011, 2013, 2015, 2017, 2018, 2020, 2021)	Year prior to depression symptoms (or closest year prior)
Education	Categorical	Primary, Secondary, Tertiary	Education was assessed with a categorization of the school leaving degree and vocational training according to the CASMIN classification. We generated one categorical variable with three categories (primary, secondary, tertiary) for this study.	Annually	Same year as depressions symptoms
Monthly income	Numeric	0+	Monthly income was assessed as equalized disposable monthly household income. We assigned the total household income to the individual level by equalizing it for household size, discounting it to the 2015 Consumer Price Index, and taking its natural logarithm.	Annually	Same year as depressions symptoms
Employment status	Categorical	Full-time, Part-time, Unemployed, Not working	Employment status was assessed with two measures of employment and occupational status. From these, we generated one categorical variable with four categories (full-time work, part-time work, unemployed, not working) for this study.	Annually	Same year as depressions symptoms
Car in household	Binary	No car in household (0), Car in household (1)	Question: ‘Which of the following apply to your household? There are one or more cars in the household’.	Irregularly (2001, 2003, 2005, 2007, 2011, 2013, 2016, 2017, 2018, 2019, 2021)	Same year as depressions symptoms (or closest year prior)
Health insurance	Binary	Public health insurance (0), Private health insurance (1)	Question: ‘What kind of health insurance do you have: statutory health insurance or are you exclusively privately insured?’	Annually	Same year as depressions symptoms
Agreeableness	Numeric	1–7	Agreeableness was assessed with three items, averaged within individuals. Example item: ‘I see myself as someone who . . .is considerate and kind to almost everyone’.^a^	Irregularly (2005, 2009, 2013, 2017, 2019, 2021)	Year prior to depression symptoms
Conscientiousness	Numeric	1–7	Conscientiousness was assessed with three items, averaged within individuals. Example item: ‘. . .tends to be lazy’ (reverse-keyed)^a^	Irregularly (2005, 2009, 2013, 2017, 2019, 2021)	Year prior to depression symptoms
Extraversion	Numeric	1–7	Extraversion was assessed with three items, averaged within individuals. Example item: ‘. . .is talkative’.^a^	Irregularly (2005, 2009, 2013, 2017, 2019, 2021)	Year prior to depression symptoms
Neuroticism	Numeric	1–7	Neuroticism was assessed with three items, averaged within individuals. Example item: ‘. . .is relaxed, handles stress well’ (reverse-keyed)^a^	Irregularly (2005, 2009, 2013, 2017, 2019, 2021)	Year prior to depression symptoms
Openness to experience	Numeric	1–7	Openness to experience was assessed with three items, averaged within individuals. Example item: ’. . .is original, comes up with new ideas’.^a^	Irregularly (2005, 2009, 2013, 2017, 2019, 2021)	Year prior to depression symptoms
Spatial access to general practitioners	Numeric	0+	Spatial access to general practitioners was generated for each individual using the 2SFCA method incorporating information on general practitioners relative to the resident population in an individual’s immediate surroundings.^b^	Annually	Same year as depressions symptoms
Spatial access to psychotherapists	Numeric	0+	Spatial access to psychotherapists was generated for each individual using the 2SFCA method, which incorporates information on psychotherapists relative to the resident population in an individual’s immediate surroundings.^b^	Annually	Same year as depressions symptoms
Season	Binary	Spring and summer (April–September = 0), Autumn and winter (October–March = 1)	The autumn and winter months were assessed by using the date of the interview when the depression symptoms were screened.	Annually	Same year as depressions symptoms
Federal state	Categorical	1–16	The federal state was captured for the current place of residence.	Annually	Same year as depressions symptoms
Urbanicity	Categorical	<2k [1], 2k–5k [2], 5k–20k [3], 20k–50k [4], 50k–100k [5], 100k–500k [6], and >500k [7]	The degree of urbanicity was captured for the current place of residence. Urbanicity was measured at the municipality level, classifying the number of inhabitants in seven categories.	Annually	Same year as depressions symptoms

*Note.* CPI = Consumer Price Index; SOEP-BFI = 15-item short form of the Big Five Inventory designed for the GSOEP; MCS score = Mental Component Summary score; PCS score = Physical Component Summary score; 2SFCA = Two-Step Floating Catchment Area method.

a The GSOEP assesses the Big Five personality traits using the SOEP-BFI, a 15-item short form of the Big Five Inventory (Gerlitz & Schupp, 2005). Each item is answered on a 7-point scale ranging from ‘strongly disagree’ (1) to ‘strongly agree’ (7). A full list of all SOEP-BFI items and corresponding traits is given in Supplemental Table S1.

b The 2SFCA method used for generating the indicators on spatial access to general practitioners and spatial access to psychotherapists is described in detail in Supplemental Note S1.

#### Depression symptoms

Depressive disorders are commonly diagnosed with structured clinical interviews (BÄK et al., 2022). However, this approach is not feasible at the population level. Fortunately, short scales with good psychometric properties are available to screen for depression, as for instance the Mental Component Summary (MCS) Score (Gill et al., 2007; Vilagut et al., 2013), which we used in this study.^1^ The MCS has been administered in the GSOEP biennially since 2002 via the SF-12v2 questionnaire. The MCS total score ranges from 1 to 100 with a score of 50 representing the mean in 2004 and higher levels indicating better mental health (Ware et al., 1998). To generate a binary indicator for depression symptoms (yes/no) reflecting the presence of 4-week depression symptoms, we relied on an MCS cutoff of 37.3 (sensitivity 86% and specificity 88% compared with a diagnostic interview; Vilagut et al., 2013).^2^

#### Formal diagnosis

To measure a formal diagnosis of a depressive disorder, we relied on respondents’ self-reports of doctors’ diagnoses, which have been included in the GSOEP biennially since 2009. Survey respondents are asked, ‘Has a doctor ever diagnosed you to have one or more of the following illnesses?’ with ‘depression’ given as a possible response.

#### Health status

We included three variables into the analyses to approximate the health status of each individual. First, to approximate depression symptom severity we included the absolute MCS score. Second, as an indicator of overall physical health status, we included the absolute Physical Component Summary score (PCS; the second subscale from the SF-12; Ware et al., 1998). Finally, we included a binary indicator reflecting the presence of at least one chronic physical condition (cancer, cardiopathy, diabetes, and stroke) in our analysis.

#### Demographics

To account for demographic characteristics, we included gender, age, and migration background.

#### Socioeconomic characteristics

To account for socioeconomic characteristics, we included variables for household composition (living alone/living together with others), number of close friends, education, monthly net income, employment status, (lack of) car in the household, and type of health insurance (private versus state health insurance).

#### Personality traits

To account for differences across personality traits, we included the Big Five personality traits of agreeableness, conscientiousness, extraversion, neuroticism, and openness to experience in our analyses (Goldberg, 1993). The GSOEP uses the SOEP-BFI, a 15-item short form of the Big Five Inventory (Gerlitz & Schupp, 2005). Supplemental Table S2 provides a complete list of the SOEP-BFI items and the corresponding traits.

#### Health infrastructure

We created two indicators of health infrastructure: spatial access to general practitioners (GPs) and spatial access to psychotherapists. To generate unique indicators for each survey respondent, we used the 2-Step Floating Catchment Area (2SFCA) method (Supplemental Note S1). The 2SFCA methodology is widely applied in health research (Stacherl & Sauzet, 2023). It measures GPs [psychotherapists] in relation to the population around an individual and assigns larger weights to GPs [psychotherapists] in closer proximity (Wang, 2012). The index is interpreted as GPs [psychotherapists] per 10,000 inhabitants with higher scores representing better spatial access.

### Statistical analyses

We investigated (1) risk factors for experiencing depression symptoms (*symptom model*) and (2) facilitators and barriers to a formal diagnosis after experiencing symptoms (*diagnosis model*). In the *symptom model*, the outcome was a binary indicator reflecting an MCS below the MCS cutoff and explanatory variables were measured in the same year as the outcome variable.^3^ In the *diagnosis model*, the outcome was a binary indicator reflecting the receipt of a first-ever formal diagnosis after experiencing symptoms and explanatory variables were taken from the year prior to outcome measurement. We used logistic regression of the following form for both models:

$$\begin{array}{l} y_{i}=\beta_{0}+\beta_{1}*health_{i}+\beta_{2}*demographic_{i}\\ \;\;\;\;\;\;\;+\beta_{3}*socioeconomic_{i}\;\;+\beta_{4}*personality_{i}\\ \;\;\;\;\;\;+\beta_{5}*infrastructure_{i}+\gamma *X_{i}+\varepsilon_{i} \end{array}$$

where $health_{i}$ reflects the individual health status, $demographic_{i}$ reflects the demographic characteristics, $socioeconomic_{i}$ reflects the socioeconomic characteristics, $personality_{i}$ reflects the Big Five personality traits, and $infrastructure_{i}$ reflects the health infrastructure. The additional term $X_{i}$ reflects controls for the season, year, federal state, and urbanicity. We applied a stepwise approach to investigate the role of each set of explanatory variables separately.

All analyses were conducted in R, version 4.2.2 (R Core Team, 2022). Data manipulation was done with the *tidyverse* package (Wickham et al., 2019), and spatial access measures were computed with the *sf* package (Pebesma, 2018).

## Results

Table 2 presents the characteristics of individuals in the *symptom sample* and the *diagnosis sample*. In the *symptom sample*, 11.0% (*N* = 7,484) screened positive for depression symptoms.^4^ In the *diagnosis sample*, 13.0% (*N* = 448) reported a first-ever formal diagnosis in the year after experiencing depression symptoms. We present results separately for the *symptom model* and the *diagnosis model* in the results section. Note that a comparative discussion of the two models will follow in the discussion section.

**Table 2.** Individual characteristics in the symptom and the diagnosis sample.

Variable	Symptom sample			Diagnosis sample		
	Total	Symptoms: no	Symptoms: yes	Total	Diagnosis: no^c^	Diagnosis: yes^d^
	*n* (%)/*m* (*SD*)	*n* (%)/*m* (*SD*)	*n* (%)/*m* (*SD*)	*n* (%)/*m* (*SD*)	*n* (%)/*m* (*SD*)	*n* (%)/*m* (*SD*)
Individuals^a^	68,222	60,738	7,484	3,444	2,996	448
2010	16,309 (23.9)	14,306 (23.6)	2,003 (26.8)	1,267 (36.8)	1,105 (36.9)	162 (36.2)
2014	2,6621 (39.0)	2,3726 (39.1)	2,895 (38.7)	933 (27.1)	829 (27.7)	104 (23.2)
2018	2,5292 (37.1)	22,706 (37.4)	2,586 (34.6)	1244 (36.1)	1,062 (35.4)	182 (40.6)
Mental health status (MCS)^b^	50.6 (9.9)	53.0 (7.2)	30.7 (5.6)	31.5 (5.1)	31.8 (4.9)	29.6 (6.0)
Physical health status (PCS)	49.4 (10.2)	49.5 (10.0)	48.1 (11.6)	48.8 (11.5)	49.2 (11.5)	45.6 (11.1)
Chronic physical condition						
No	36,146 (65.8)	32513 (66.3)	3,633 (61.7)	2,245 (65.2)	1,966 (65.6)	279 (62.3)
Yes	18,765 (34.2)	16,510 (33.7)	2,255 (38.3)	1,199 (34.8)	1,030 (34.4)	169 (37.7)
Age (years)^a^						
<30	11,254 (16.5)	9,927 (16.3)	1,327 (17.7)	572 (16.6)	508 (17.0)	64 (14.3)
30–44	17,923 (26.3)	15,825 (26.1)	2,098 (28.0)	893 (25.9)	793 (26.5)	100 (22.3)
45–59	19,872 (29.1)	17,527 (28.9)	2,345 (31.3)	1,041 (30.2)	862 (28.8)	179 (40.0)
60–74	13,393 (19.6)	12,312 (20.3)	1,081 (14.4)	574 (16.7)	498 (16.6)	76 (17.0)
75+	5,780 (8.5)	5,147 (8.5)	633 (8.5)	364 (10.6)	335 (11.2)	29 (6.5)
Gender^a^						
Male	31,658 (46.4)	28,909 (47.6)	2,749 (36.7)	1,356 (39.4)	1,208 (40.3)	148 (33.0)
Female	36,564 (53.6)	31,829 (52.4)	4,735 (63.3)	2,088 (60.6)	1,788 (59.7)	300 (67.0)
Migration background						
No	57,471 (84.2)	51,102 (84.1)	6,369 (85.1)	3,027 (87.9)	2,632 (87.9)	395 (88.2)
Yes	10,751 (15.8)	9,636 (15.9)	1,115 (14.9)	417 (12.1)	364 (12.1)	53 (11.8)
Household composition^a^						
Living with others	57,903 (84.9)	51,902 (85.5)	6,001 (80.2)	2,776 (80.7)	2,417 (80.8)	359 (80.1)
Living alone	10,319 (15.1)	8,836 (14.5)	1,483 (19.8)	665 (19.3)	576 (19.2)	89 (19.9)
Number of close friends^b^	4.1 (3.6)	4.2 (3.6)	3.5 (3.2)	3.6 (3.4)	3.7 (3.5)	3.2 (2.8)
Education^a^						
Primary	7,648 (11.6)	6,564 (11.1)	1,084 (15.0)	404 (12.1)	348 (12.0)	56 (13.0)
Secondary	42,292 (63.9)	37,564 (63.8)	4,728 (65.3)	2,229 (66.8)	1,934 (66.5)	295 (68.4)
Tertiary	16,195 (24.5)	14,769 (25.1)	1426 (19.7)	706 (21.1)	626 (21.5)	80 (18.6)
Employment status^a^						
Full-time	27,737 (40.7)	25181 (41.5)	2,556 (34.2)	1318 (38.3)	1,170 (39.1)	148 (33.0)
Part-time	140,88 (20.7)	12,591 (20.7)	1,497 (20.0)	662 (19.2)	564 (18.8)	98 (21.9)
Unemployed	3,206 (4.7)	2,517 (4.1)	689 (9.2)	220 (6.4)	167 (5.6)	53 (11.8)
Not working	23,191 (34.0)	20,449 (33.7)	2,742 (36.6)	1244 (36.1)	1,095 (36.5)	149 (33.3)
Monthly income^b^	1173.8 (728.1)	1190.6 (739.4)	1037.8 (611.9)	1097.7 (629.7)	1101.9 (620.3)	1070.1 (689.0)
Car in household^a^						
No	8,762 (14.6)	7,395 (13.7)	1,367 (21.3)	588 (17.6)	496 (17.1)	92 (21.1)
Yes	51,434 (85.4)	46,387 (86.3)	5,047 (78.7)	2750 (82.4)	2,406 (82.9)	344 (78.9)
Health insurance^a^						
Public	59,579 (87.4)	52,806 (87.1)	6,773 (90.6)	3,062 (89.0)	2,667 (89.1)	395 (88.2)
Private	8556 (12.6)	7853 (12.9)	703 (9.4)	379 (11.0)	326 (10.9)	53 (11.8)
Agreeableness^b^	5.4 (1.0)	5.4 (1.0)	5.2 (1.0)	5.2 (1.0)	5.2 (1.0)	5.2 (1.0)
Conscientiousness^b^	5.8 (0.9)	5.8 (0.9)	5.6 (1.0)	5.6 (1.0)	5.6 (1.0)	5.6 (1.0)
Extraversion^b^	4.8 (1.1)	4.9 (1.1)	4.6 (1.2)	4.7 (1.2)	4.7 (1.2)	4.6 (1.2)
Neuroticism^b^	3.8 (1.2)	3.7 (1.2)	4.6 (1.2)	4.4 (1.2)	4.4 (1.2)	4.8 (1.2)
Openness to experience^b^	4.6 (1.2)	4.6 (1.2)	4.5 (1.3)	4.5 (1.3)	4.5 (1.2)	4.4 (1.3)
Spatial access to GPs^b^	6.7 (5.5)	6.7 (5.5)	6.7 (5.5)	6.7 (5.6)	6.6 (5.6)	6.9 (5.4)
Spatial access to psychotherapists^b^	2.8 (3.0)	2.8 (3.0)	2.9 (3.0)	2.8 (3.0)	2.7 (3.0)	2.9 (3.0)

*Note.* MCS = Mental Component Summary score; PCS = Physical Component Summary score.

a Categorical variables: The number of observations per category is displayed with the corresponding percentage in parentheses.

b Continuous variables: The mean is displayed with the standard deviation in parentheses.

### Risk factors for depression symptoms

Table 3 presents the findings on risk factors associated with depression symptoms. Regarding *health status*, having a chronic physical condition was positively associated with depression symptoms, OR = 1.243, 95% CI [1.162, 1.329].

**Table 3.** Regression results, symptom model: predictors of depression symptoms.

Variable	Model 1		Model 2		Model 3		Model 4		Model 5	
	*OR*	95% CI	*OR*	95% CI	*OR*	95% CI	*OR*	95% CI	*OR*	95% CI
Physical health status (PCS)	0.889	[0.867, 0.912]	0.838	[0.816, 0.861]	0.910	[0.885, 0.936]	0.985	[0.957, 1.015]	0.985	[0.956, 1.014]
Chronic physical condition	1.123	[1.058, 1.192]	1.427	[1.336, 1.525]	1.370	[1.283, 1.463]	1.242	[1.161, 1.328]	1.243	[1.162, 1.329]
Gender: Female [Male]			1.556	[1.480, 1.636]	1.493	[1.415, 1.576]	1.294	[1.221, 1.373]	1.295	[1.222, 1.373]
Age: 30–44 [<30]			0.904	[0.839, 0.974]	1.076	[0.994, 1.164]	1.202	[1.106, 1.307]	1.202	[1.106, 1.307]
Age: 45–59 [<30]			0.796	[0.737, 0.859]	0.979	[0.901, 1.063]	1.164	[1.068, 1.269]	1.164	[1.068, 1.269]
Age: 60–75 [<30]			0.438	[0.397, 0.482]	0.452	[0.408, 0.501]	0.591	[0.531, 0.658]	0.592	[0.531, 0.659]
Age: 75+ [<30]			0.538	[0.479, 0.605]	0.480	[0.423, 0.545]	0.687	[0.602, 0.783]	0.688	[0.603, 0.784]
Migration Background: Yes [No]			0.893	[0.832, 0.959]	0.751	[0.697, 0.810]	0.752	[0.694, 0.815]	0.753	[0.694, 0.816]
Household: Living alone [Living with others]					1.320	[1.230, 1.417]	1.405	[1.307, 1.511]	1.403	[1.305, 1.510]
Number of close friends					0.793	[0.765, 0.822]	0.866	[0.836, 0.897]	0.866	[0.836, 0.897]
Education: Primary [Secondary]					1.060	[0.981, 1.145]	1.015	[0.936, 1.101]	1.017	[0.938, 1.103]
Education: Tertiary [Secondary]					0.966	[0.901, 1.036]	0.939	[0.874, 1.010]	0.937	[0.871, 1.007]
Employment: Part-time [Full-time]					0.954	[0.886, 1.027]	0.873	[0.808, 0.943]	0.872	[0.807, 0.942]
Employment: Unemployed [Full-time]					1.794	[1.614, 1.995]	1.572	[1.406, 1.757]	1.572	[1.406, 1.758]
Employment: Not working [Full-time]					1.376	[1.276, 1.484]	1.207	[1.115, 1.306]	1.206	[1.114, 1.305]
Monthly income					0.733	[0.685, 0.785]	0.781	[0.730, 0.836]	0.779	[0.728, 0.834]
Car in household: Yes [No]					0.882	[0.818, 0.952]	0.898	[0.825, 0.978]	0.900	[0.826, 0.981]
Health insurance: Private [Public]					0.997	[0.912, 1.090]	0.999	[0.912, 1.095]	0.999	[0.911, 1.095]
Agreeableness							0.912	[0.886, 0.940]	0.913	[0.886, 0.940]
Conscientiousness							0.878	[0.852, 0.905]	0.878	[0.852, 0.905]
Extraversion							0.879	[0.854, 0.905]	0.879	[0.854, 0.905]
Neuroticism							1.930	[1.868, 1.995]	1.930	[1.868, 1.995]
Openness to experience							1.030	[0.997, 1.064]	1.029	[0.996, 1.063]
Spatial access to GPs									0.985	[0.958, 1.012]
Spatial access to psychotherapists									1.019	[0.987, 1.052]
Constant	0.112	[0.093, 0.134]	0.105	[0.087, 0.127]	0.791	[0.481, 1.301]	0.429	[0.260, 0.708]	0.440	[0.266, 0.727]
Season-fixed effects^a^	Yes		Yes		Yes		Yes		Yes	
Year-fixed effects^a^	Yes		Yes		Yes		Yes		Yes	
Federal state-fixed effects^a^	Yes		Yes		Yes		Yes		Yes	
Urbanicity-fixed effects^a^	Yes		Yes		Yes		Yes		Yes	
N observations	68,222		68,222		68,222		68,222		68,222	
AIC	46,945		46,246		45,299		42,362		42,364	
McFadden *R*²	.006		.022		.042		.104		.104	

*Note.* AIC = Akaike Information Criterion; CI = confidence interval; *OR* = odds ratio.

a Season, survey year, federal state, and urbanicity were controlled for. Coefficients are omitted for table clarity.

For *demographics*, female gender was positively associated with depression symptoms, OR = 1.295, 95% CI [1.222, 1.373], age was non-linearly associated with depression symptoms. Specifically, age groups 30 to 44 and 45 to 59 years were positively associated with depression symptoms: OR = 1.202, 95% CI [1.106, 1.307], and OR = 1.164, 95% CI [1.068, 1.269]; whereas age groups 60 to 74 and 75+ years were negatively associated with depression symptoms, OR = 0.592, 95% CI [0.531, 0.659], and OR = 0.688, 95% CI [0.603, 0.784]. Finally, migration background was negatively associated with depression symptoms, OR = 0.753, 95% CI [0.694, 0.816].

For = *socioeconomic characteristics*, living alone (OR = 1.403, 95% CI [1.305, 1.510]), being unemployed (OR = 1.572, 95% CI [1.406, 1.758]), and not working (OR = 1.206, 95% CI [1.114, 1.305]) were positively associated with depression symptoms, whereas the number of close friends (OR = 0.866, 95% CI [0.836, 0.897]), working part-time (OR = 0.872, 95% CI [0.807, 0.942]), income (OR = 0.779, 95% CI [0.728, 0.834]), and having a car in the household (OR = 0.900, 95% CI [0.826, 0.981]) were negatively associated with depression symptoms.

For the Big Five *personality traits*, neuroticism was positively associated, OR = 1.930, 95% CI [1.868, 1.995], and agreeableness, conscientiousness, and extraversion were negatively associated with depression symptoms (agreeableness: OR = 0.913, 95% CI [0.886, 0.940]; conscientiousness: OR = 0.878, 95% CI [0.852, 0.905]; and extraversion: OR = 0.879, 95% CI [0.854, 0.905]). All other predictors, that is the overall physical health status, level of education, having a private health insurance and spatial access to general practitioners and psychotherapists were not associated with depression symptoms.

### Facilitators and barriers to receiving a formal diagnosis

Table 4 presents the findings on factors associated with receiving a depression diagnosis after experiencing depression symptoms.

**Table 4.** Regression results, diagnosis model: predictors of formal diagnosis.

Variable	Model 1		Model 2		Model 3		Model 4		Model 5	
	*OR*	95% CI	*OR*	95% CI	*OR*	95% CI	*OR*	95% CI	*OR*	95% CI
Mental health status (MCS)	0.674	[0.616, 0.738]	0.664	[0.606, 0.728]	0.665	[0.606, 0.730]	0.669	[0.609, 0.735]	0.668	[0.608, 0.734]
Physical health status (PCS)	0.683	[0.611, 0.763]	0.624	[0.553, 0.705]	0.629	[0.553, 0.716]	0.641	[0.563, 0.730]	0.639	[0.561, 0.728]
Chronic physical condition	0.865	[0.684, 1.096]	1.065	[0.826, 1.372]	1.068	[0.826, 1.381]	1.048	[0.810, 1.357]	1.040	[0.803, 1.347]
Gender: Female [Male]			1.391	[1.117, 1.732]	1.376	[1.087, 1.742]	1.330	[1.041, 1.699]	1.331	[1.042, 1.701]
Age: 30–44 [<30]			0.941	[0.665, 1.333]	0.896	[0.627, 1.281]	0.92	[0.639, 1.324]	0.921	[0.640, 1.326]
Age: 45–59 [<30]			1.325	[0.946, 1.856]	1.247	[0.878, 1.772]	1.318	[0.920, 1.886]	1.331	[0.930, 1.907]
Age: 60–74 [<30]			0.743	[0.488, 1.131]	0.771	[0.496, 1.201]	0.806	[0.514, 1.264]	0.811	[0.517, 1.272]
Age: 75+ [<30]			0.329	[0.192, 0.563]	0.353	[0.198, 0.632]	0.364	[0.201, 0.659]	0.368	[0.203, 0.665]
Migration Background: Yes [No]			0.795	[0.572, 1.105]	0.800	[0.568, 1.127]	0.803	[0.569, 1.134]	0.799	[0.566, 1.128]
Household: Living alone [Living with others]					1.166	[0.870, 1.563]	1.198	[0.892, 1.610]	1.184	[0.880, 1.591]
Number of close friends					0.874	[0.763, 1.000]	0.91	[0.795, 1.041]	0.908	[0.793, 1.039]
Education: Primary [Secondary]					0.934	[0.661, 1.321]	0.928	[0.654, 1.317]	0.931	[0.656, 1.322]
Education: Tertiary [Secondary]					0.951	[0.701, 1.290]	0.972	[0.713, 1.323]	0.964	[0.707, 1.314]
Employment: Part-time [Full-time]					1.233	[0.906, 1.680]	1.204	[0.882, 1.643]	1.197	[0.877, 1.635]
Employment: Unemployed [Full-time]					1.864	[1.228, 2.829]	1.830	[1.202, 2.787]	1.831	[1.202, 2.787]
Employment: Not working [Full-time]					0.935	[0.670, 1.304]	0.904	[0.645, 1.266]	0.908	[0.648, 1.272]
Monthly income					0.997	[0.756, 1.315]	1.032	[0.781, 1.364]	1.038	[0.784, 1.373]
Car in household: Yes [No]					0.989	[0.719, 1.359]	1.028	[0.745, 1.419]	1.050	[0.759, 1.452]
Health insurance: Private [Public]					1.433	[1.006, 2.041]	1.374	[0.962, 1.962]	1.368	[0.958, 1.953]
Agreeableness							0.978	[0.876, 1.092]	0.978	[0.876, 1.091]
Conscientiousness							0.946	[0.846, 1.057]	0.946	[0.846, 1.057]
Extraversion							0.988	[0.880, 1.110]	0.985	[0.877, 1.107]
Neuroticism							1.278	[1.144, 1.428]	1.281	[1.147, 1.432]
Openness to experience							0.944	[0.841, 1.059]	0.945	[0.842, 1.061]
Spatial access to GPs									1.067	[0.952, 1.197]
Spatial access to psychotherapists									1.032	[0.904, 1.177]
Constant	0.041	[0.016, 0.108]	0.031	[0.011, 0.087]	0.029	[0.003, 0.248]	0.020	[0.002, 0.178]	0.020	[0.002, 0.176]
Season-fixed effects^a^	Yes		Yes		Yes		Yes		Yes	
Year-fixed effects^a^	Yes		Yes		Yes		Yes		Yes	
Federal state-fixed effects^a^	Yes		Yes		Yes		Yes		Yes	
Urbanicity-fixed effects^a^	Yes		Yes		Yes		Yes		Yes	
N observations	3,444		3,444		3,444		3,444		3,444	
AIC	2,560		2,517		2,515		2,501		2,502	
McFadden *R*²	.059		.080		.088		.098		.098	

*Note.* AIC = Akaike Information Criterion; CI = Confidence Interval; *OR* = odds ratio.

a Season, survey year, federal state, and urbanicity were controlled for. Coefficients are omitted for table clarity.

Regarding *health status*, overall mental health as well as overall physical health were negatively associated with receiving a formal diagnosis, that is, a worse health status made receiving a diagnosis more likely; mental health: OR = 0.668, 95% CI [0.608, 0.734] and physical health: OR = 0.639, 95% CI [0.561, 0.728]. For *demographics*, female gender was positively associated with receiving a formal diagnosis, 1.331, 95% CI [1.042, 1.701], and age was non-linearly associated with receiving a diagnosis, with elderly individuals (75 and above) being less likely to receive one, OR = 0.368, 95% CI [0.203, 0.665]. For *socioeconomic characteristics*, being unemployed was positively associated with receiving a formal diagnosis, OR = 1.831, 95% CI [1.202, 2.787]. Among the *personality traits*, only neuroticism was positively associated with receiving a diagnosis, OR = 1.281, 95% CI [1.147, 1.432]. Thus, neither the presence of a chronic physical condition, having a direct migration background, living alone, the number of friends, education, income, employment status, having a car in the household, or having private health insurance was associated with receiving a diagnosis once symptoms were present. Further, also agreeableness, conscientiousness, extraversion, and openness were not associated with receiving a diagnosis after experiencing symptoms. Finally, regarding the *health infrastructure*, neither spatial access to general practitioners nor to psychotherapists was associated with receiving a diagnosis after experiencing depression symptoms.

## Discussion

### Risk factors for depression symptoms

The present research largely replicates existing findings on risk factors for depression symptoms (e.g. female gender, middle age, living alone, low income, high neuroticism) in a large population-representative German sample, the GSOEP (Hammen & Watkins, 2018; Remes et al., 2021). These findings corroborate the use of representative panel studies to assess population-wide risk factors for mental health conditions. Further, in this study, we employed the SF-12v2 to create a binary indicator to screen for the presence of depression symptoms. Since no validated cutoffs existed for the MCS in the SF-12v2, we established one based on previous prevalence estimates of depression symptoms in Western Europe using the SF-12v1 (Vilagut et al., 2013). The consistency of prevalence estimates across studies and time (Hapke et al., 2019; Streit et al., 2023) and the fact that using an MCS cutoff of 37.3 identified similar risk factors as former studies (Lorant, 2003; Maske et al., 2016), provides initial evidence supporting the validity of this new cutoff value in the SF-12v2.

### Facilitators and barriers to receiving a formal diagnosis

We found that individuals with poorer mental and physical health, women, middle-aged and younger individuals, unemployed individuals, and individuals with higher levels of neuroticism were more likely to receive a formal diagnosis following depression symptoms. Conversely, individuals with better health, men, older individuals, employed or retired individuals, and those with lower levels of neuroticism appeared to encounter some barriers in receiving a diagnosis. These facilitators and barriers may reflect differences in how individuals perceive, seek, access, and utilize healthcare services (Levesque et al., 2013). For instance, individuals with poorer physical health tend to visit their doctors more frequently (Hajek et al., 2017), increasing the likelihood of receiving a depression diagnosis during these visits. Further, research indicates that men and women exhibit divergent perceptions of healthcare needs (Johnson & Whisman, 2013), express symptoms differently (Martin et al., 2013), and engage in distinct healthcare-seeking behaviors, possibly influenced by gendered attitudes toward mental health issues (Möller-Leimkühler, 2002).

Unemployed individuals frequently receive less social support from their social networks (Krug et al., 2022) compared to employed individuals. To compensate for this lack of support, they may rely more on institutionalized support systems like doctors, rendering them more likely to receive a formal depression diagnosis once symptoms are present (Li et al., 2023).

More neurotic individuals have more difficulty self-regulating (Yang et al., 2020) and tend to worry more in general, potentially rendering them more likely to visit a doctor and share their symptoms (Seekles et al., 2012). Interestingly, although research from other health systems has outlined a relationship between the distance from health services and the likelihood of depression treatment (Desai et al., 2023), this finding does not seem to hold in Germany. We found that the health infrastructure does not pose a barrier for receiving a diagnosis in Germany. This might be explained by an overall high level of access to health services in Germany, which has one of the highest densities of physicians in Europe (Blümel et al., 2020).

**Figure 1.**
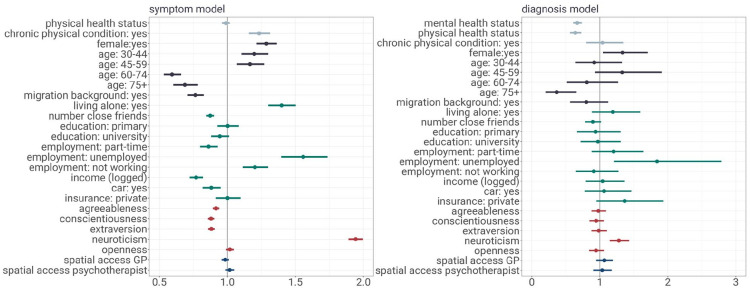
The figure shows regression results on a symptom model and a diagnosis model. The symptom model examines risk factors for depression symptoms (outcome: binary indicator for MCS below cutoff) in a logistic regression for the full sample. The diagnosis model examines determinants of receiving a diagnosis of depression (outcome: binary indicator of first-ever depression diagnosis) in a logistic regression for the sample experiencing prior depression symptoms. Estimates are reported as odds ratios with 95% confidence intervals.

Figure 1 depicts both the *symptom model* and *diagnosis model* results, highlighting the convergence and disparities of factors associated with depression symptoms and with receiving a depression diagnosis. Interestingly, when comparing predictors of depression symptoms and formal diagnosis, several factors emerge as both risk factors for experiencing symptoms and as facilitators for receiving a diagnosis. Women, younger and middle-aged individuals, unemployed individuals, and individuals with higher levels of neuroticism are more prone to experiencing depression symptoms. However, once symptoms manifest, individuals with these characteristics are also more likely to be diagnosed, even when considering symptom severity. This finding is important for studies relying on depression diagnosis data to examine risk factors for depressive disorders. Failing to acknowledge these factors as facilitators of diagnosis may lead to an overemphasis on certain risk factors’ impact on depression prevalence. Moreover, this finding suggests that individuals with these characteristics are more likely to be identified as depressive in medical practice. This aligns with research showing physicians’ tendency to better detect depressive episodes in women compared to men (Potts et al., 1991) and younger adults compared to older individuals (Mitchell et al., 2010). Variation in diagnostic outcomes may partly stem from a focus on ‘typical’ patients who exhibit ‘typical’ risk factors (e.g. women, young to middle-aged adults, those with high neuroticism). This focus is shaped by both medical and psychosocial models (Pilecki, 2020).^5^ Both in the medical model and in a psychosocial approach, female gender (biological factors), young and middle age, unemployment (life course factors), and neuroticism (psychological factors) correspond to characteristics of typical depression patients. Thus, even with similar symptom presentation, individuals ticking the boxes of a standard patient may be issued a diagnosis more promptly.

Furthermore, our findings reveal subtle differences between risk factors for depression symptoms and factors that facilitate a formal diagnosis. Specifically, physical health influences the process through two distinct pathways: While having a chronic physical condition was associated with depression symptoms but not with receiving a formal diagnosis, poorer overall physical health was linked to receiving a diagnosis but not to the emergence of depression symptoms. These results imply that the psychological burden of a chronic physical condition is more closely tied to the onset of depression symptoms, whereas the general state of physical health is more closely related to the frequency of medical consultations and, consequently, the likelihood of receiving a diagnosis.

### Limitations and future research

We wish to address three limitations of this study. First, due to the longitudinal design, participants were required to engage in the GSOEP for three consecutive years. Consequently, the most severe cases of depression may have been underrepresented, as these individuals might have discontinued their survey participation due to their symptoms or treatment. To address the potential influence of symptom severity on the variables of interest, we included a symptom severity indicator. This indicator revealed that individuals with higher symptom severity were indeed more likely to receive a diagnosis. Future research should therefore aim to cover the entire spectrum of depression severity.

Second, based on our data we are unable to assess the clinical relevance of a depression diagnosis or its absence. Receiving a depressive disorder diagnosis does not necessarily benefit all individuals who meet diagnostic criteria. Concerns about overdiagnosis persist (Mojtabai, 2013; Parker, 2007), as this can lead to the medicalization of depressive episodes that may not require clinical intervention (Horwitz & Wakefield, 2023). Conversely, evidence also points to underdiagnosis in cases that would indeed benefit from clinical intervention (Hickie, 2007). While this limitation does not directly affect our interpretation, further research on the clinical relevance of depression diagnoses and underlying factors thereof is essential.

Lastly, in our study, we were unable to track whether individuals received treatment after the diagnosis. Yet, factors influencing diagnosis may differ from those influencing receiving treatment. For instance, while depressive disorders are often diagnosed in general practice, treatment availability typically relies on psychotherapists, who may have limited availability. To guide health policy decisions effectively, it is crucial to explore the facilitators and barriers to depression treatment post-diagnosis. Thus, future research should investigate the pathway from formal diagnosis to depression treatment.

## Conclusion

This study provides two key findings: First, it replicates previous international research on various risk factors for depression symptoms using a large representative sample from the German population. Second, it distinguishes between factors predicting a diagnosis and those contributing to symptoms. Female gender, middle age, unemployment, and higher neuroticism emerged as both risk factors for depression symptoms and facilitators for receiving a diagnosis. These results suggest that certain factors associated with depression symptoms may also aid in the diagnostic process.

## Supplemental Material

sj-docx-1-isp-10.1177_00207640241303038 – Supplemental material for From feeling depressed to getting diagnosed: Determinants of a diagnosis of depression after experiencing symptoms Supplemental material, sj-docx-1-isp-10.1177_00207640241303038 for From feeling depressed to getting diagnosed: Determinants of a diagnosis of depression after experiencing symptoms by Barbara Stacherl and Theresa M Entringer in International Journal of Social Psychiatry

## Notes

1. Another instrument for screening depression symptoms in the GSOEP is the Patient Health Questionnaire 2 (PHQ-2), which assesses two primary symptoms of major depression over the past 2 weeks. However, we opted for the MCS in this study due to the significant reduction in sample size that would have resulted from using the PHQ-2 (*N* = 1,752 compared to *N* = 3,444). This is because only one wave of the PHQ-2 data could have been used in this study, compared to the three waves available for the MCS. Nonetheless, to test for the robustness of our findings across different screening instruments, we conducted additional analyses using the PHQ-2 to screen for depression symptoms (Kroenke et al., 2003). The results of these analyses can be found in Supplemental Note S2, Supplemental Table S3, and Supplemental Table S4.

2. Comparing the MCS from the SF-12v1 with a standard diagnostic interview, Vilagut et al. (2013) determined an optimal MCS cutoff at 45.6, yielding a sensitivity of 86% and a specificity of 88%. Relying on this optimal cutoff as a screener, they estimated a depression symptom prevalence of 13.5% in 2002 in Western Europe. Using the MCS in the GSOEP (based on the SF-12v2), we were able to reproduce this 13.5% depression symptom prevalence estimate for Germany in 2002 with a GSOEP-specific MCS cutoff of 37.3. We used this GSOEP-specific MCS cutoff to generate the binary depression symptom indicator.

3. To avoid issues of reverse causality, personality traits and the number of close friends were assessed from the year prior to measuring depression symptoms. This approach accounts for potential changes in these factors and their perception that might occur as a consequence of experiencing depression symptoms.

4. In detail, estimates of the prevalence of 4-week depression symptoms in the German population were 13.4%, 95% CI [12.6, 14.2] in 2010, 11.6%, 95% CI [11.0, 12.2] in 2014, and 10.6%, 95% CI [10.0, 11.2] in 2018. Prevalence was estimated with frequency weights, resulting in estimates representative of the German population. Our prevalence estimates were very similar to estimates which rely on the Patient-Health Questionnaire as a screening tool in the German population. These 2-week depression symptom prevalence estimates ranged from 7.8% to 10.1% in 2014 (Hapke et al., 2019; Streit et al., 2023).

5. The medical model views mental disorders as chemical imbalances that cause specific symptoms defining a disorder, while the psychosocial model attributes mental illness to interpersonal, cultural, and societal factors. Thus, the medical model emphasizes biological markers and brain chemistry, whereas the psychosocial model focuses on contextual factors underlying mental health issues (Pilecki, 2020).
