# Supplementary material for: From feeling depressed to getting diagnosed: Determinants of a diagnosis of depression after experiencing symptoms
Source: Int J Soc Psychiatry. 2024 Dec 26;71(4):723–37. doi: 10.1177/00207640241303038 (PMC12171077; doi:10.1177/00207640241303038)
Supplement: sj-docx-1-isp-10.1177_00207640241303038 – Supplemental material for From feeling depressed to getting diagnosed: Determinants of a diagnosis of depression after experiencing symptoms [file sj-docx-1-isp-10.1177_00207640241303038.docx]

**Supplemental Materials**

From feeling depressed to getting diagnosed:

Determinants of a diagnosis of depression after experiencing symptoms

Barbara Stacherl^1^ & Theresa M. Entringer^1, 2^

^1^ German Institute for Economic Research (DIW Berlin), Mohrenstraße 58, 11017 Berlin, Germany

^2^ University of Greifswald, Department of Psychology, Franz-Mehring Straße 47, 17489 Greifswald

**Supplemental Figure S1**

*Theorized Pathway from Depression Symptoms to Formal Diagnosis*


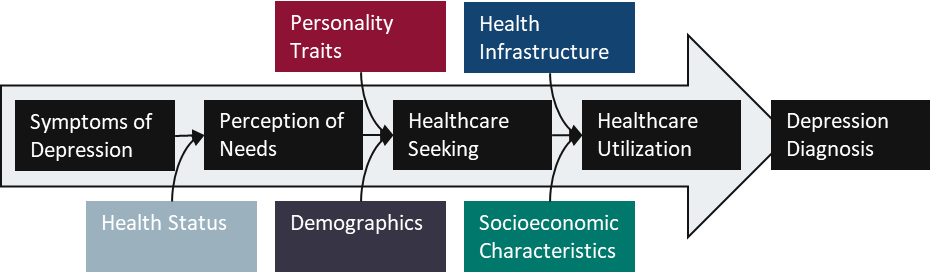


*Note.* Pathway based on “Conceptual framework for access to health care” (Levesque, Harris, & Russell, 2013)

**Supplemental Figure S2**

*Time of Assessment of Measures Used in Analyses*


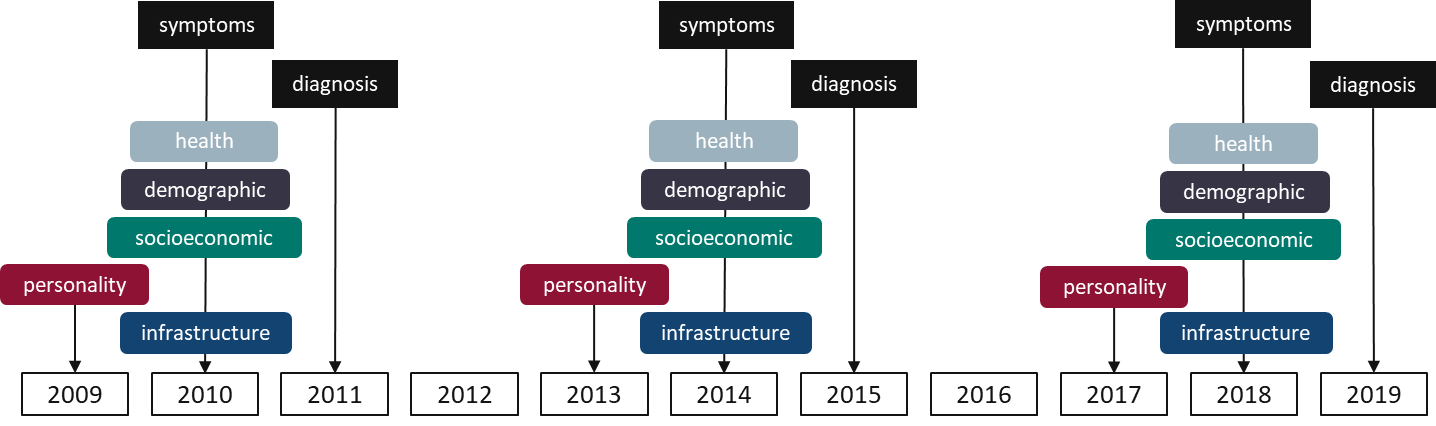


**Supplemental Table S1**

*List of the SOEP-BFI Items to Measure the Big Five Personality Traits*

| I am… | Variable | Big Five trait |
| --- | --- | --- |
| a thorough worker | plh0212 | Conscientiousness |
| communicative, talkative | plh0213 | Extraversion |
| sometimes somewhat rude to others | plh0214 | Agreeableness |
| original, someone who comes up with new ideas | plh0215 | Openness |
| a worrier | plh0216 | Neuroticism |
| forgiving | plh0217 | Agreeableness |
| somewhat lazy (r) | plh0218 | Conscientiousness |
| outgoing, sociable | plh0219 | Extraversion |
| someone who values artistic, aesthetic experiences | plh0220 | Openness |
| nervous | plh0221 | Neuroticism |
| effective and efficient in completing tasks | plh0222 | Conscientiousness |
| reserved (r) | plh0223 | Extraversion |
| considerate and kind to others | plh0224 | Agreeableness |
| imaginative | plh0225 | Openness |
| relaxed, able to deal with stress (r) | plh0226 | Neuroticism |

*Note:* The GSOEP assesses the Big Five personality traits using the SOEP-BFI, a 15-item short form of the Big Five Inventory (Gerlitz & Schupp, 2005). Each item is answered on a 7-point scale ranging from “strongly disagree” (1) to “strongly agree” (7).

**Supplemental Note S1**

*Operationalization of Spatial Access to Healthcare*

To operationalize an individual’s spatial access to healthcare we used a two-stage floating catchment area (2SFCA) method. 2SFCA measures are widely used in health research to quantify spatial healthcare access. The intuition is similar to a physician density in that it captures provider-to-population ratios, however, it has two main advantages: First, it is not a regional aggregate but depicts spatial access at a specific location and thus also accounts for potential border crossing in healthcare use. Second, it integrates availability and accessibility by incorporating both the provider-to-population ratio and distance decay (decreasing access with increasing distance) effects. The 2SFCA method was first presented by Luo & Wang (2003) and consists of two steps:

1. Around each provider location $j$, all population locations $k$ within a predefined catchment area (e.g., 30 minutes driving time) are searched. The provider capacity (e.g., number of physicians) is divided by the total population within the catchment area to create a location-specific provider-to-population ratio. This takes on the form:

$$R_{j}= \frac{S_{j}}{\sum_{k \in\{d_{kj}\leq d_{o}\}} P_{k}}$$

where$R_{j}$ is the provider-to-population ratio at location $j$, $S_{j}$ is the provider capacity at location $j$, $P_{k}$ is the population at location $k$, $d$ is the distance between healthcare provider and population location, and $d_{0}$ is the distance threshold defining the catchment area.

1. Around each population location $i$, all provider locations $j$ within the same predefined catchment area are searched. The provider-to-population ratios of all provider locations within the catchment area are summed up to build the spatial access measure. This takes on the form:

$$A_{i}= \sum_{j \in\{d_{ij}\leq d_{o}\}} R_{j} =\sum_{j \in\{d_{ij}\leq d_{o}\}} \frac{S_{j}}{\sum_{k \in\{d_{kj}\leq d_{o}\}} P_{k}}$$

Since its initial introduction, many methodological advancements have been proposed to the 2SFCA method. Most notably, continuous (instead of binary) distance decay functions have been introduced to reflect varying accessibility also within the catchment area. Wang (2012) proposed a generalized 2SFCA method which adds a generic distance decay function $f(d)$ in both steps. This study built on the generalized 2SFCA method, applying a negative power function, more specifically, an inverse distance decay function. This took on the form:

$$A_{ip}= \sum_{j \in\{d_{ij}\leq d_{o}\}} \frac{S_{j} d_{ij}^{-\beta}}{\sum_{k \in\{d_{kj}\leq d_{o}\}} P_{k} d_{kj}^{-\beta}}$$

where $A_{ip}$ is the spatial access at location $i$ for provider type $p$, $S_{j}$ is the capacity (here: full-time equivalents) of the healthcare provider at location $j$, $P_{k}$ is the population at location $k$, $d$ is the distance between healthcare provider and population location, and $d^{-\beta}$ reflects the negative power distance decay function. In our study the friction coefficient $\beta$ took on a value of 1, thus the distance decay function corresponded to an inverse distance weighting. We assumed equal travel friction for all distances below 100m to avoid extreme inverse distance weights for very close proximity (e.g., doctor’s practice at respondent address).

This measure was applied once for access to psychotherapists with $d_{0}$ = 10km and once for access to general practitioners with $d_{0}$ = 3km. The catchment area definition was based on the Euclidean (aerial) distance. We relied on small-area level census data (100mx100m grid) to depict the population distribution. We defined the provider-to-population ratio per 10,000 inhabitants. We used census data from the year 2011 as this was the only year in our study period for which the fine-grained population data were available. Current provider data for general practitioner and psychotherapist locations (geocoded street addresses) were used for 2009-2019. Thus, we computed for each individual in our study sample the spatial access to general practitioners as well as the spatial access to psychotherapists for each year. The 2SFCA for general practitioners is to be interpreted as follows: Number of general practitioner full-time equivalents per 10,000 inhabitants within a 3km radius around the individual, demand-adjusted and distance weighted. The 2SFCA for psychotherapists is to be interpreted as follows: Number of psychotherapist full-time equivalents per 10,000 inhabitants within a 10km radius around the individual, demand-adjusted and distance weighted.

**Supplemental Table S2**

*Number of Observations per Person and Year by Sample Restrictions and Total Number of Observations Aggregated Over Time*

|  | Number of Persons who… | | | Final Number of |  |
| --- | --- | --- | --- | --- | --- |
| Base Year ^a^ | Screened Positive for Symptoms | Participated in Year Prior and After | Had No Prior Diagnosis | Person-Unique Observations | |
| 2010 | 2,215 | 1,707 | 1,418 | 1,267 | |
| 2014 | 2,926 | 1,488 | 1,107 | 933 | |
| 2018 | 2,612 | 1,909 | 1,358 | 1,244 | |
| Sum | 7,753 | 5,104 | 3,883 | 3,444 | |

*Note*^.a^ Base year refers to the year in which depression symptoms were assessed.

**Supplemental Note S2**

*Sensitivity Analyses*

To test for the robustness of our findings across different tools for screening depression symptoms, we repeated the analyses using the Patient Health Questionnaire (PHQ-2). This two-item measure assesses the two key depressive symptoms over the past two weeks: “feeling down, depressed, or hopeless” and “little interest or pleasure in doing things”. Responses range from “not at all” (0) to “almost every day” (3), with the PHQ-2 score derived from the sum of these values. We employed a PHQ-2 cutoff of 3 to identify the presence of current depression symptoms (Kroenke, Spitzer, & Williams, 2003). Although the PHQ-2 was available in three waves of the GSOEP (2016, 2019, 2021), our longitudinal study design limited its use to one wave (2016).^[[1]](#footnote-1)^ Consequently, sample sizes were smaller when using the PHQ-2. Specifically, the PHQ-2 symptom sample consisted of N = 24,112 (compared to *N* = 68,222 in the MCS symptom sample) and a PHQ-2 diagnosis sample consisted of N = 1,752 persons (compared to *N* = 3,444 in the MCS diagnosis sample). As can be seen in Table S3 and S4, most of our findings replicated across the two samples. However, some findings also became non-significant when using the PHQ-2 instead of the MCS and in a few rare occasions effects also emerged when using the PHQ-22 instead of the MCS. In detail:

1. Regarding risk factors for depression symptoms, chronic physical conditions, living alone, primary education, unemployment, income, private health insurance, agreeableness, conscientiousness, extroversion, neuroticism, spatial access to general practitioners, and spatial access to psychotherapists were consistently associated with depression symptoms in the PHQ-2 and the MCS symptom sample. Differences in the significance of effects were noted, however, for physical health status, age, gender, migration background, tertiary education, part-time work, not working, and car in household. While the association between depression symptoms and age, gender, migration background, part-time work, not working, and car in the household remained largely the same but became non-significant when using the PHQ-2 instead of the MCS; an association between depression symptoms and physical health status, and tertiary education appeared when using the PHQ-2 instead of the MCS.
2. Regarding facilitators and barriers to diagnosis, there was substantial agreement in the direction of effects across the two screening instruments. However, most effects became non-significant in the PHQ-2 diagnosis sample compared to the MCS diagnosis sample. However, only overall mental and physical health status were significantly linked to diagnosis likelihood in the PHQ-2 analysis, while gender, age, unemployment, and neuroticism became non-significant when using the PHQ-2 instead of the MCS.

There are at least three reasons why the results differed somewhat depending on the depression screening instruments used. First, the sample sizes varied significantly between the instruments, with the PHQ-2 resulting in much smaller sample sizes compared to the MCS. This smaller sample size for the PHQ-2 reduces its statistical power to detect significant effects. Second, the PHQ-2 consists of only 2 items, whereas the MCS includes a larger number of items. Consequently, the PHQ-2 has lower reliability than the MCS, making it inherently more challenging to detect significant effects with the PHQ-2. Linally, the samples were collected at different times, introducing the possibility of period effects influencing the results. However, future research is necessary to gain a better understanding of these differences.

**Supplemental Table S3**

*Regression Results, Symptom Model: Predictors of Depression Symptoms (Depression Symptoms Screened based on PHQ-2 ≥ 3)*

|  | Model 1 | | Model 2 | | Model 3 | | Model 4 | | Model 5 | |
| --- | --- | --- | --- | --- | --- | --- | --- | --- | --- | --- |
|  | OR | 95% CI | OR | 95% CI | OR | 95% CI | OR | 95% CI | OR | 95% CI |
| Physical health status (PCS) | 0.632 | [0.607, 0.658] | 0.589 | [0.564, 0.614] | 0.635 | [0.608, 0.663] | 0.660 | [0.630, 0.690] | 0.660 | [0.630, 0.690] |
| Chronic physical condition | 1.120 | [1.015, 1.235] | 1.454 | [1.305, 1.620] | 1.391 | [1.248, 1.551] | 1.321 | [1.182, 1.476] | 1.320 | [1.182, 1.475] |
| Gender: Female [Male] |  |  | 1.185 | [1.092, 1.286] | 1.143 | [1.047, 1.249] | 1.038 | [0.940, 1.146] | 1.037 | [0.939, 1.146] |
| Age: 30–44 [<30] |  |  | 0.721 | [0.635, 0.819] | 0.850 | [0.744, 0.971] | 0.882 | [0.768, 1.014] | 0.883 | [0.768, 1.014] |
| Age: 45–59 [<30] |  |  | 0.625 | [0.549, 0.712] | 0.756 | [0.660, 0.867] | 0.798 | [0.689, 0.924] | 0.798 | [0.689, 0.924] |
| Age: 60–74 [<30] |  |  | 0.368 | [0.313, 0.432] | 0.439 | [0.370, 0.521] | 0.498 | [0.414, 0.598] | 0.498 | [0.414, 0.598] |
| Age: 75+ [<30] |  |  | 0.409 | [0.340, 0.492] | 0.448 | [0.366, 0.548] | 0.535 | [0.431, 0.663] | 0.535 | [0.431, 0.663] |
| Migration Background: Yes [No] |  |  | 1.067 | [0.957, 1.189] | 0.915 | [0.816, 1.025] | 0.909 | [0.805, 1.026] | 0.908 | [0.805, 1.025] |
| Household: Living alone [Living with others] |  |  |  |  | 1.302 | [1.158, 1.463] | 1.344 | [1.193, 1.514] | 1.344 | [1.193, 1.514] |
| Number of close friends |  |  |  |  | 0.885 | [0.839, 0.934] | 0.942 | [0.896, 0.991] | 0.942 | [0.896, 0.991] |
| Education: Primary [Secondary] |  |  |  |  | 1.094 | [0.965, 1.241] | 1.056 | [0.915, 1.219] | 1.056 | [0.914, 1.219] |
| Education: Tertiary [Secondary] |  |  |  |  | 0.855 | [0.759, 0.963] | 0.846 | [0.751, 0.953] | 0.846 | [0.751, 0.954] |
| Employment: Part-time [Full-time] |  |  |  |  | 0.965 | [0.854, 1.089] | 0.904 | [0.798, 1.024] | 0.904 | [0.798, 1.024] |
| Employment: Unemployed [Full-time] |  |  |  |  | 1.562 | [1.317, 1.852] | 1.437 | [1.202, 1.718] | 1.437 | [1.202, 1.718] |
| Employment: Not working [Full-time] |  |  |  |  | 1.064 | [0.937, 1.209] | 0.955 | [0.838, 1.088] | 0.955 | [0.838, 1.089] |
| Monthly income |  |  |  |  | 0.689 | [0.618, 0.769] | 0.714 | [0.640, 0.796] | 0.714 | [0.641, 0.797] |
| Car in household: Yes [No] |  |  |  |  | 0.892 | [0.792, 1.003] | 0.908 | [0.802, 1.029] | 0.908 | [0.802, 1.030] |
| Health insurance: Private [Public] |  |  |  |  | 0.881 | [0.748, 1.038] | 0.892 | [0.756, 1.053] | 0.892 | [0.756, 1.054] |
| Agreeableness |  |  |  |  |  |  | 0.949 | [0.903, 0.998] | 0.949 | [0.903, 0.998] |
| Conscientiousness |  |  |  |  |  |  | 0.923 | [0.879, 0.969] | 0.923 | [0.879, 0.969] |
| Extraversion |  |  |  |  |  |  | 0.892 | [0.844, 0.943] | 0.892 | [0.844, 0.943] |
| Neuroticism |  |  |  |  |  |  | 1.533 | [1.438, 1.635] | 1.534 | [1.438, 1.635] |
| Openness to experience |  |  |  |  |  |  | 1.013 | [0.953, 1.076] | 1.013 | [0.953, 1.077] |
| Spatial access to GPs |  |  |  |  |  |  |  |  | 1.007 | [0.965, 1.052] |
| Spatial access to psychotherapists |  |  |  |  |  |  |  |  | 0.995 | [0.944, 1.050] |
| Constant | 0.121 | [0.092, 0.160] | 0.147 | [0.109, 0.197] | 1.758 | [0.803, 3.852] | 1.378 | [0.637, 2.980] | 1.369 | [0.632, 2.965] |
| Season-fixed effects ^a^ | Yes | | Yes | | Yes | | Yes | | Yes | |
| Federal state-fixed effects ^a^ | Yes | | Yes | | Yes | | Yes | | Yes | |
| Urbanicity-fixed effects ^a^ | Yes | | Yes | | Yes | | Yes | | Yes | |
| N observations | 24,112 | | 24,112 | | 24,112 | | 24,112 | | 24,112 | |
| AIC | 16810 | | 16632 | | 16346 | | 15828 | | 15832 | |
| McFadden R² | 0.043 | | 0.053 | | 0.071 | | 0.101 | | 0.101 | |

*Note.* AIC=Akaike Information Criterion; CI=Confidence Interval; OR=Odds Ratio.
^a^ Season, federal state, and urbanicity were controlled for. Coefficients are omitted for table clarity.

**Supplemental Table S4**

*Regression Results, Diagnosis Model: Predictors of Formal Diagnosis (Depression Symptoms Screened Based on PHQ-2 ≥ 3)*

|  | Model 1 | | Model 2 | | Model 3 | | Model 4 | | Model 5 | |
| --- | --- | --- | --- | --- | --- | --- | --- | --- | --- | --- |
|  | OR | 95% CI | OR | 95% CI | OR | 95% CI | OR | 95% CI | OR | 95% CI |
| Mental health status (MCS) | 0.426 | [0.354, 0.513] | 0.413 | [0.342, 0.500] | 0.421 | [0.346, 0.512] | 0.409 | [0.332, 0.503] | 0.406 | [0.330, 0.500] |
| Physical health status (PCS) | 0.760 | [0.631, 0.915] | 0.703 | [0.576, 0.858] | 0.734 | [0.594, 0.908] | 0.734 | [0.594, 0.907] | 0.726 | [0.588, 0.898] |
| Chronic physical condition | 1.034 | [0.711, 1.504] | 1.286 | [0.864, 1.914] | 1.197 | [0.800, 1.791] | 1.213 | [0.806, 1.827] | 1.208 | [0.801, 1.824] |
| Gender: Female [Male] |  |  | 1.020 | [0.718, 1.449] | 1.001 | [0.686, 1.461] | 0.946 | [0.629, 1.422] | 0.967 | [0.641, 1.460] |
| Age: 30–44 [<30] |  |  | 1.234 | [0.683, 2.230] | 1.329 | [0.726, 2.434] | 1.290 | [0.696, 2.389] | 1.339 | [0.720, 2.488] |
| Age: 45–59 [<30] |  |  | 1.287 | [0.720, 2.303] | 1.480 | [0.808, 2.711] | 1.433 | [0.767, 2.674] | 1.517 | [0.808, 2.849] |
| Age: 60–74 [<30] |  |  | 0.712 | [0.342, 1.479] | 0.655 | [0.301, 1.427] | 0.648 | [0.290, 1.451] | 0.700 | [0.311, 1.576] |
| Age: 75+ [<30] |  |  | 0.410 | [0.177, 0.950] | 0.334 | [0.133, 0.834] | 0.349 | [0.135, 0.904] | 0.402 | [0.153, 1.053] |
| Migration Background: Yes [No] |  |  | 1.295 | [0.824, 2.033] | 1.270 | [0.792, 2.035] | 1.242 | [0.766, 2.016] | 1.239 | [0.762, 2.015] |
| Household: Living alone [Living with others] |  |  |  |  | 1.026 | [0.629, 1.673] | 1.013 | [0.617, 1.664] | 0.966 | [0.587, 1.590] |
| Number of close friends |  |  |  |  | 1.103 | [0.921, 1.321] | 1.063 | [0.886, 1.276] | 1.054 | [0.875, 1.268] |
| Education: Primary [Secondary] |  |  |  |  | 0.844 | [0.508, 1.400] | 0.870 | [0.518, 1.461] | 0.857 | [0.510, 1.440] |
| Education: Tertiary [Secondary] |  |  |  |  | 0.996 | [0.594, 1.672] | 0.968 | [0.572, 1.637] | 0.912 | [0.537, 1.548] |
| Employment: Part-time [Full-time] |  |  |  |  | 1.030 | [0.601, 1.766] | 1.041 | [0.605, 1.792] | 1.026 | [0.595, 1.769] |
| Employment: Unemployed [Full-time] |  |  |  |  | 1.152 | [0.595, 2.233] | 1.120 | [0.564, 2.225] | 1.106 | [0.556, 2.198] |
| Employment: Not working [Full-time] |  |  |  |  | 1.489 | [0.864, 2.567] | 1.477 | [0.843, 2.588] | 1.431 | [0.815, 2.513] |
| Monthly income |  |  |  |  | 0.872 | [0.536, 1.420] | 0.853 | [0.513, 1.417] | 0.823 | [0.494, 1.373] |
| Car in household: Yes [No] |  |  |  |  | 0.642 | [0.396, 1.043] | 0.627 | [0.381, 1.033] | 0.654 | [0.395, 1.083] |
| Health insurance: Private [Public] |  |  |  |  | 1.131 | [0.543, 2.357] | 1.143 | [0.543, 2.407] | 1.103 | [0.521, 2.337] |
| Agreeableness |  |  |  |  |  |  | 1.021 | [0.827, 1.260] | 1.026 | [0.827, 1.273] |
| Conscientiousness |  |  |  |  |  |  | 1.022 | [0.780, 1.340] | 1.014 | [0.780, 1.318] |
| Extraversion |  |  |  |  |  |  | 1.161 | [0.895, 1.507] | 1.156 | [0.887, 1.507] |
| Neuroticism |  |  |  |  |  |  | 0.992 | [0.789, 1.248] | 0.989 | [0.778, 1.257] |
| Openness to experience |  |  |  |  |  |  | 1.148 | [0.821, 1.606] | 1.165 | [0.832, 1.632] |
| Spatial access to GPs |  |  |  |  |  |  |  |  | 0.964 | [0.791, 1.175] |
| Spatial access to psychotherapists |  |  |  |  |  |  |  |  | 1.282 | [1.054, 1.559] |
| Constant | 0.099 | [0.032, 0.306] | 0.079 | [0.023, 0.268] | 0.243 | [0.008, 7.305] | 0.298 | [0.009, 9.727] | 0.421 | [0.013, 13.999] |
| Season-fixed effects ^a^ | Yes | | Yes | | Yes | | Yes | | Yes | |
| Federal state-fixed effects ^a^ | Yes | | Yes | | Yes | | Yes | | Yes | |
| Urbanicity-fixed effects ^a^ | Yes | | Yes | | Yes | | Yes | | Yes | |
| N observations | 1752 | | 1752 | | 1752 | | 1752 | | 1752 | |
| AIC | 1027 | | 1023 | | 1032 | | 1035 | | 1033 | |
| McFadden R² | 0.116 | | 0.131 | | 0.140 | | 0.146 | | 0.152 | |

*Note.* AIC=Akaike Information Criterion; CI=Confidence Interval; OR=Odds Ratio.
^a^ Season, federal state, and urbanicity were controlled for. Coefficients are omitted for table clarity.

1. In our longitudinal study design, we required to measure depression diagnosis once *before* measuring depression symptoms (to include only individuals starting out without depression diagnosis) and once *after* measuring depression symptoms to capture the outcome (first-ever depression diagnosis in year after). In the GSOEP, depression diagnosis data are surveyed in odd years. Hence, we could apply our longitudinal study design with the PHQ-2 measuring depression symptoms only for the year 2016, not for the years 2019 and 2021. [↑](#footnote-ref-1)
